# Inhibition of Spinal Interlukin-33/ST2 Signaling and Downstream ERK and JNK Pathways in Electroacupuncture Analgesia in Formalin Mice

**DOI:** 10.1371/journal.pone.0129576

**Published:** 2015-06-12

**Authors:** Ping Han, Shenbin Liu, Mengting Zhang, Jing Zhao, Yanqing Wang, Gencheng Wu, Wenli Mi

**Affiliations:** 1 Department of Integrative Medicine and Neurobiology, School of Basic Medical Sciences, State Key Laboratory of Medical Neurobiology, Institutes of Brain Science, Collaborative Innovation Center for Brain Science, Fudan University, Shanghai, China; 2 Neuroscience and Neuroengineering Center, Med-X Research Institute Shanghai Jiao Tong University, Shanghai, China; University of São Paulo, BRAZIL

## Abstract

Although acupuncture is widely used to manage pain, it remains highly controversial, largely due to the lack of a clear mechanism for its benefits. Here, we investigated the role of IL-33, a novel interleukin (IL)-1 family member, and its receptor ST2 in the analgesic effects of electroacupuncture (EA) on formalin-induced inflammatory pain. The results showed that 1) EA stimulation of ipsilateral Zusanli (ST 36) and Yanglingquan (GB 34) acupoints for 30 min remarkably suppressed the two phases of formalin-induced spontaneous pain; 2) subcutaneous or intrathecal administration of recombinant IL-33 (rIL-33) significantly inhibited the analgesic effect of EA, whereas the ST2 antibody potentiated EA analgesia in formalin mice; 3) EA treatment decreased the up-regulation of IL-33 and ST2 protein following formalin injection; and 4) the suppression of the formalin-induced expression of spinal phosphorylated ERK and JNK induced by EA treatment was significantly attenuated following subcutaneous rIL-33 delivery, and was further decreased by the ST2 antibody. These data suggest that EA alleviates formalin-induced inflammatory pain, at least partially, by inhibiting of spinal IL-33/ST2 signaling and the downstream ERK and JNK pathways.

## Introduction

Acupuncture, a widely used method in traditional medicine in China and other Asian countries for thousands of years, has been confirmed to be effective in pain relief by numerous clinical observations and experimental studies [1–3]. Electroacupuncture (EA) is a modified acupuncture technique utilizing electrical stimulation that is being actively studied for objective control over stimulating parameters. Despite its modulatory effect on the expression and release of various endogenous bioactive substances, including opioids, monoamines, adenosine, neurotrophins, and cytokines [4–7], the precise mechanism of action of EA analgesia is still not clearly understood. Works from our laboratory and from others have shown that cytokines, such as interleukin (IL)-1β, IL-6 and tumor necrosis factor (TNF)-α, was involved in the mechanism of action of EA analgesia in inflammatory pain [8,9].

IL-33, which was identified as a member of the IL-1 family in 2005, has attracted considerable attention since its discovery [10]. In addition to its function as a stored alarmin and the unraveling property of its transcriptional or gene regulatory function in the nucleus, IL-33 plays a critical role in the defense against a wide range of pathogens and mediates a broad range of diseases, including infectious, inflammatory, autoimmune, and cardiovascular diseases [11–14]. IL-33 exerts its cytokine function in a classical receptor-mediated fashion by binding to a receptor complex consisting of ST2 and the IL-1 receptor accessory protein (IL-1RAcP), initiating a signaling cascade similar to the cascade initiated by IL-1 and IL-18 [14].

Previous studies have shown that peripheral IL-33 mediated antigen-induced cutaneous and articular hypernociception in mice [15–17]. Our recent studies demonstrated that spinal IL-33 and its receptor ST2 mediated formalin-induced acute inflammatory pain and 4T1 carcinoma cells inoculation-induced chronic bone cancer pain in mice [18,19]. Whether IL-33 and its receptor ST2 are regulated in EA analgesia during inflammatory pain has not yet been examined.

IL-33 binds to ST2 and activates downstream signaling pathways, such as mitogen-activated protein kinases (MAPKs), nuclear factor-κB (NF-κB), and Janus kinase 2 (JAK2) [10,20,21]. The activation of MAPK pathways in the spinal dorsal horn plays an important role in central sensitization and pain modulation [22]. Additionally, it has been reported that the analgesic effect of acupuncture is mediated by the inhibition of MAPK activation (including ERK, p38 MAPK, and JNK) in various pain conditions [23–26]. Therefore, whether the downstream effects of IL-33/ST2 signaling during EA treatment are also mediated by MAPK pathways needs to be investigated.

Using a classic mouse model of acute inflammatory pain based on the intra-plantar injection of formalin, the present study was performed to assess the modulation of spinal IL-33 and its receptor ST2 in EA analgesia and to further examine its subsequent downstream MAPK signaling pathways.

## Materials and Methods

### Animals

Experiments were performed on adult male BALB/c mice aged 7–9 weeks and weighing 20–25 g; the mice were supplied by the Experimental Animal Center, Chinese Academy of Sciences, Shanghai. Prior to experimental manipulation, the mice were allowed to acclimate for one week in groups of four mice per cage and maintained under controlled conditions (22 ± 1°C, 6 a.m. to 6 p.m. alternate light-dark cycles) with access to food and water ad libitum. All experiments were conducted strictly in accordance with the National Institutes of Health Guide for the Care and Use of Laboratory Animals and the guidelines of the International Association for the Study of Pain [27], as well as the Animal Research Welfare Council of School of Basic Medical Science of Fudan University (20140226–086). All efforts were made to minimize the number of animals used and their suffering.

### Formalin test

The formalin test was examined, as previously described [18]. Briefly, mice were acclimated in Plexiglas chambers (15 cm×12 cm×10 cm) for at least 30 min before the experiments. After injecting with 20 μl of 5% formalin (in normal saline) into the right hind paw plantar, the mice immediately returned to the behavioral chamber. And then the lifting and licking duration of the injected paw every 5 min during the 45-min observation period was monitored. In order to observe the injected paw clearly, a mirror was positioned below the behavioral chamber at a 45° angle. All the testing was performed blinded.

### Electroacupuncture treatment

**Fig 1:**
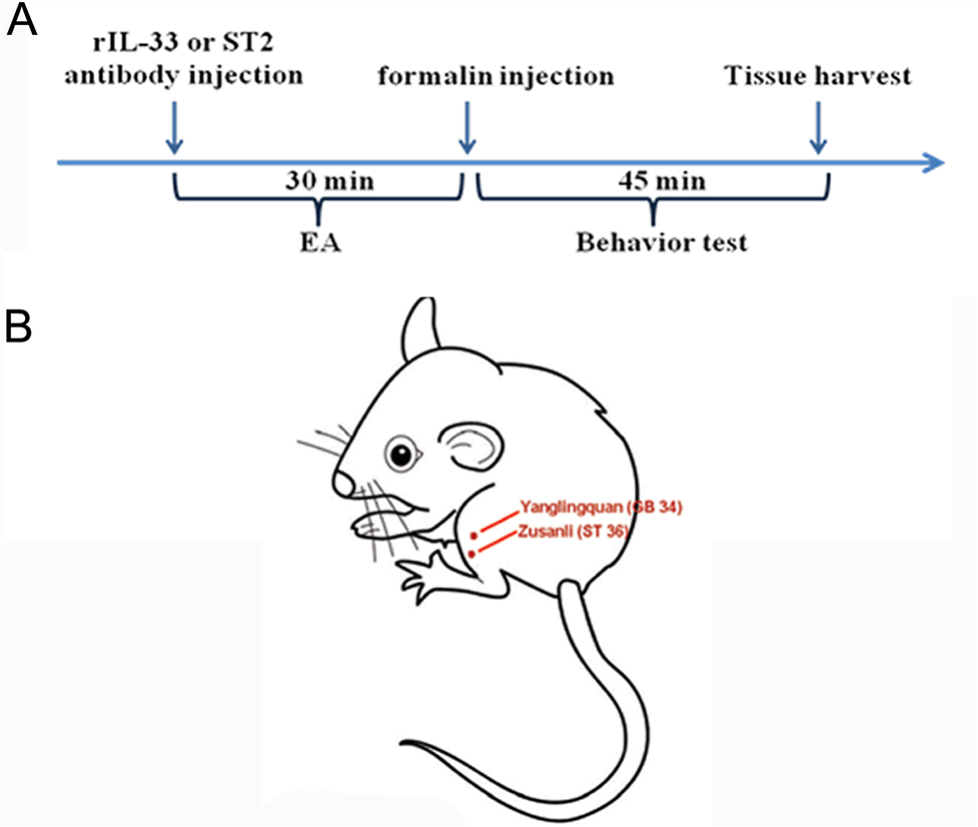
Outline of the experimental protocol and electroacupuncture (EA) stimulation. (A) Mice received EA or sham EA for 30 min after subcutaneous or intrathecal administration of recombinant IL-33 (rIL-33) or the ST2 antibody, and then formalin was immediately administered via intra-plantar injection. Paw lifting and licking duration were observed for 45 min. After the behavior test, the spinal cord was harvested. (B) Schematic diagram showing the two acupoints applied to the formalin mice. Acupuncture was ipsilaterally applied at two specific acupoints, Zusanli (ST 36) and Yanglingquan (GB 34), throughout the experiments.

The detailed EA procedure has been described previously [28]. In brief, during EA treatment, the trunk of the mouse was kept motionless while the head and four limbs maintained freedom of movement in a specially designed holder. The mice were allowed to acclimate for 30 min before EA treatment. The skin was cleaned with alcohol swabs, and a pair of stainless steel needles with diameters of 0.3 mm were inserted into the ipsilateral acupoints ‘Zusanli’ (ST 36, located on the anterior tibia muscle in approximately the upper 1/6 of the length of the lower leg below the knee) and ‘Yanglingquan’ (GB 34, located near the knee joint anterior and inferior to the small head of the fibula in the muscle peroneus longus and brevis, where the common peroneal nerve bifurcates into the superficial and deep peroneal nerves) at a depth of 2 and 3 mm, respectively, as shown in Fig 1B. The two needles were connected with the output terminals of an EA apparatus (the HANS Acupoint Nerve Stimulator, LH202H, Beijing, China). Alternating strains of dense-sparse frequencies (2/100 Hz) were selected. The intensity of the stimulation was approximately 1–2 mA, and mild muscle twitching was observed. The stimulation lasted for 30 min, and then formalin was injected. The sham EA group of animals underwent the same manipulation as the EA group without the electrical current during treatment. Briefly, after the skin was cleaned, a pair of the needles same as EA treatment was inserted into the ipsilateral acupoints at the same depth, similar to EA group for 30 min. But the needles were not connected with the EA apparatus.

ST36 and GB34 were chosen based on TCM meridian theory and their common use in previous studies in the treatment of inflammatory pain in both clinical and basic research [2,3,8,29].

### Drug administration

The mouse ST2 antibody and normal goat IgG were dissolved in sterile phosphate buffer solution (PBS), and mouse recombinant IL-33 (rIL-33) was dissolved in sterile PBS containing 1% bovine serum albumin. All drugs were purchased from R&D Systems, USA. ST2 antibody or rIL-33 was administered either subcutaneously into the right hind paw or intrathecally before EA treatment, that is, 30 min before the formalin injection, as shown in Fig 1A. The control groups were administered normal goat IgG or sterile PBS, respectively.

The intrathecal injection was conducted via lumbar puncture, as previously described [18]. Briefly, the mice were anesthetized with isoflurane. Then, the drugs (5 μl in volume) were injected into the subarachnoid space of lumbar vertebrae L5 and L6 with a 5-μl syringe (Hamilton, 33-gauge needle). A tail flick indicated that the needle had pierced the dura.

### Western blot analysis

The lumbar spinal cord was removed and homogenized in sample buffer (0.01 M Tris–HCl buffer (pH 7.6) containing 0.25 M sucrose, 0.1 M NaCl, 1 mM EDTA and 1 mM phenylmethyl sulfonylfluoride) at 4°C. After 12,000 r.p.m. centrifugation for 10 min, the supernatant was used for western blot analysis. Samples (30 μg of total protein) were dissolved with equal volume of loading buffer, separated via 10% SDS-PAGE and then electro-transferred at 300 mA to Immuno-Blot PVDF membranes for 1 h. Membranes were blocked in TBST containing 5% non-fat milk overnight at 4°C before incubation for 1 h at room temperature with goat anti-IL-33(1:1000, R&D Systems, USA), goat anti-ST2 (1:1000, R&D Systems, USA), rabbit anti-p-ERK 1/2 polyclonal antibody (1:1000, Cell Signaling Technology, USA), rabbit anti-p-p38 MAPK polyclonal antibody (1:500, Cell Signaling Technology, USA), rabbit anti-p-JNK1/2 polyclonal antibody (1:1000, Cell Signaling Technology, USA), rabbit anti-ERK 1/2 polyclonal antibody (1:1000, Cell Signaling Technology, USA), rabbit anti-p38 MAPK polyclonal antibody (1:1000, Cell Signaling Technology, USA), rabbit anti-JNK1/2 polyclonal antibody (1:1000, Cell Signaling Technology, USA) or anti-β-actin antibody (1:10000, Cell Signaling Technology, USA), diluted in TBST containing 5% BSA. The blots were washed extensively in TBST and incubated with goat-anti-rabbit IgG or goat-anti-mouse IgG conjugated to horseradish peroxidase (1:10000, Vector, USA) in TBST/1.25% BSA for 1 h at room temperature. The signal was detected by an enhanced chemiluminescence method (ECL kit, Pierce, USA), western blot images were captured on an ImageQuant LAS4000 mini image analyzer (GE Healthcare, Buckinghamshire, UK), and the band levels were quantified using Image J software, version 1.42q.

### Statistical analysis

Data were presented as the mean ± the standard error of the mean (SEM), and all statistical analyses were performed using SPSS 19.0 statistical software (SPSS Inc., Chicago, IL). For multiple comparisons, one-way analysis of variance (ANOVA) was used, which was followed by the least-significant difference test to compare different treatment groups. Student t-tests were used when two treatment groups were compared. In all statistical analyses, P<0.05 was considered the criteria for significance.

## Results

### Analgesic effect of EA on formalin-induced pain behavior in mice

**Fig 2:**
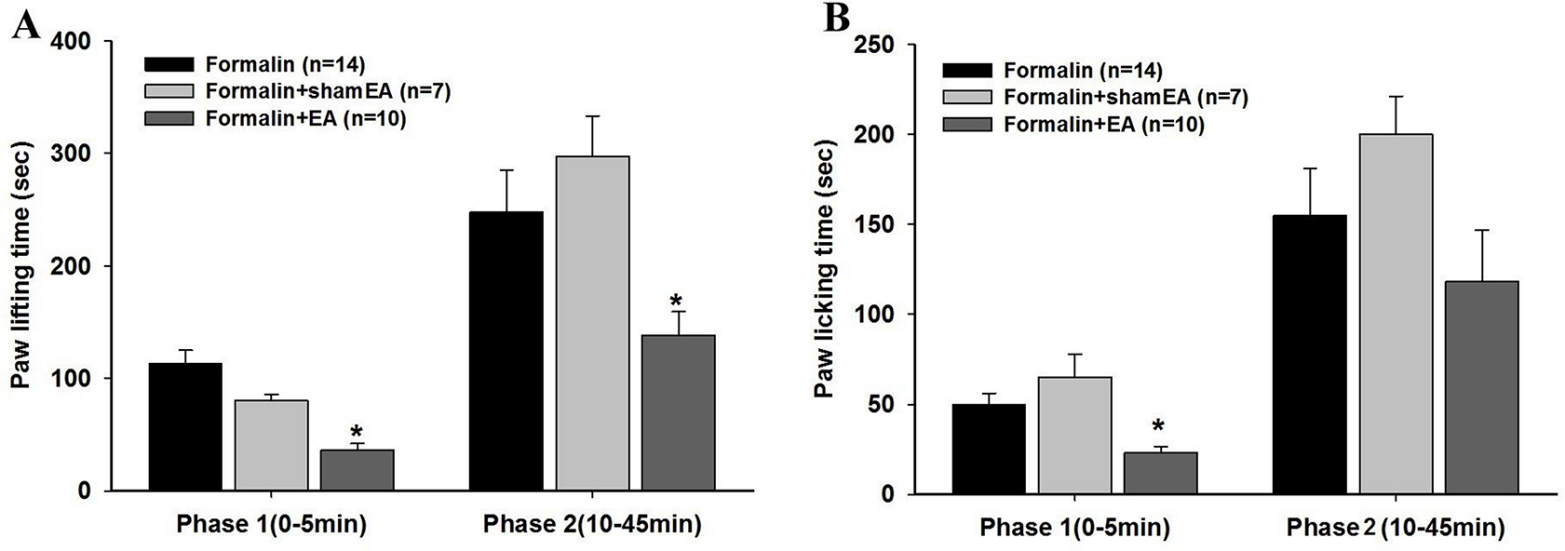
Effects of electroacupuncture (EA) on formalin-induced pain in mice. EA was applied to ST 36 and GB 34 for 30 min before the formalin injection. The paw lifting time (A) and the paw licking time (B) were statistically decreased by EA pretreatment. Data are presented as the mean ± S.E.M. *p < 0.05 vs. the formalin group.

EA was applied to ST 36 and GB 34 for 30 min before the formalin injection. Compared with the formalin group, pretreatment with EA significantly decreased the paw lifting time in the two phases following the injection when (P < 0.05) (Fig 2), while there was no significant change in the sham EA group (P > 0.05, Fig 2A). Additionally, EA remarkably reduced the paw licking time in phase 1 after the formalin injection (P < 0.05, Fig 2B).

### Recombinant IL-33 blocked the EA analgesic effect on formalin-induced pain

**Fig 3:**
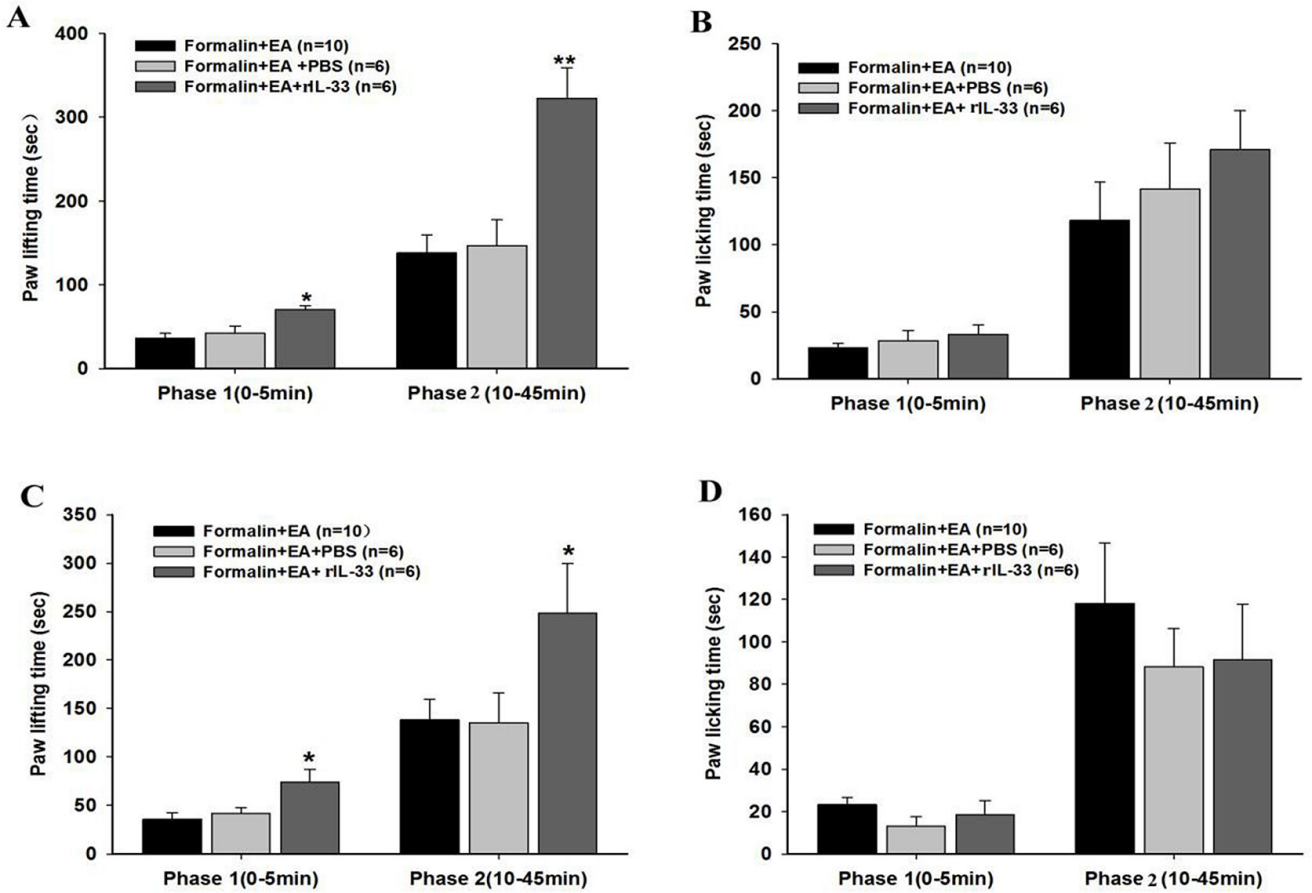
Effects of recombinant IL-33 (rIL-33) on electroacupuncture (EA) analgesia in formalin mice. EA was applied to ST 36 and GB 34 for 30 min before the formalin injection. Recombinant IL-33 was given either subcutaneously (A, B) or intrathecally (C, D) immediately before EA treatment, and paw lifting (A, C) and licking (B, D) times were recorded. Data are presented as the mean ± S.E.M. *p < 0.05 vs. the Formalin+EA+PBS group.

To observe the effect of IL-33 on EA analgesia on formalin-induced pain, rIL-33 was given either subcutaneously (300 ng) or intrathecally (3 ng) before EA treatment. After EA treatment for 30 min, mice were injected with formalin, and the spontaneous pain behavior was then tested. The subcutaneous administration of 300 ng of rIL-33 markedly increased paw lifting but not licking time in both phases in EA treatment mice compared with the Formalin+EA+PBS vehicle group (Fig 3A and 3B), indicating that IL-33 blocked the EA analgesic effect in the formalin mice. Similarly, intrathecal injection of rIL-33 significantly increased paw lifting time in the two phases but showed no significant influence on licking time (P < 0.05) (Fig 3C and 3D).

### The ST2 antibody potentiated the EA analgesic effect on formalin-induced inflammatory pain

**Fig 4:**
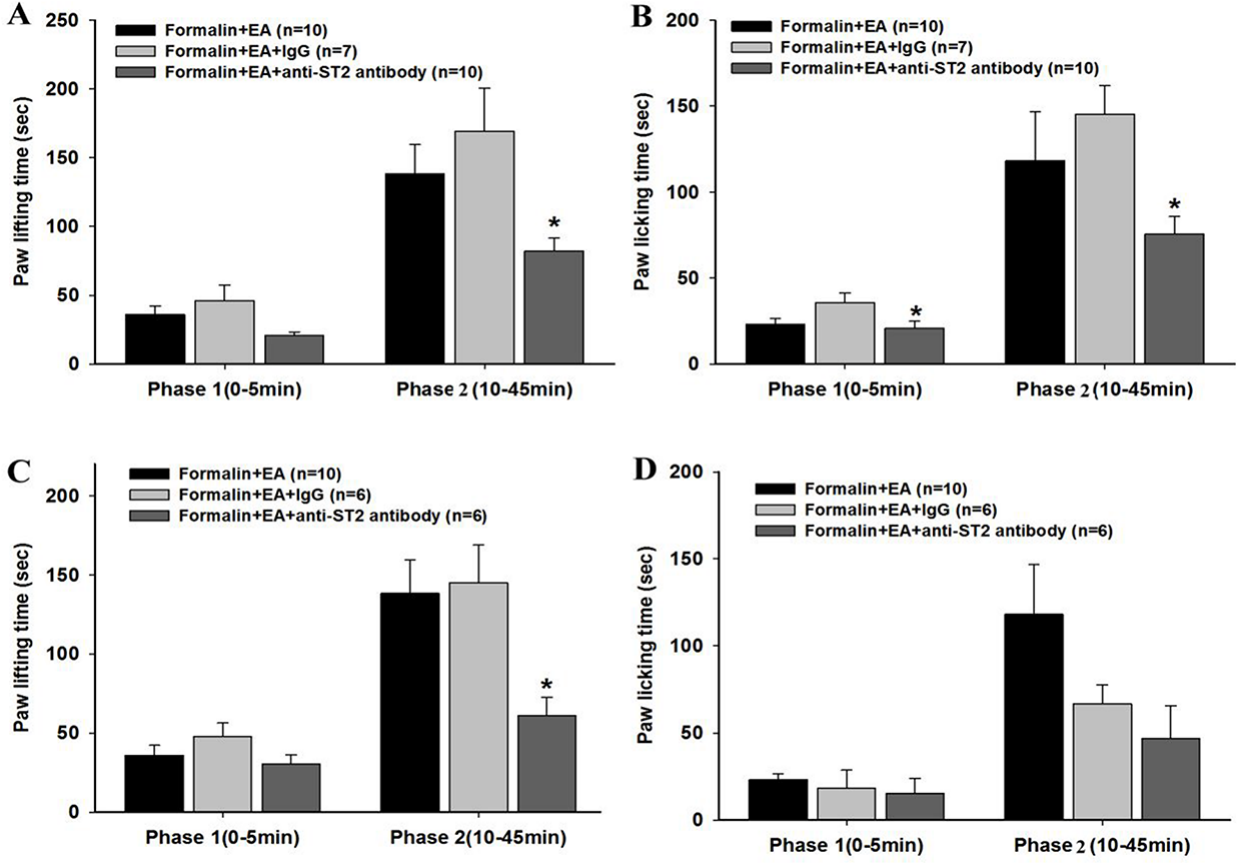
Effects of ST2 antibody on electroacupuncture (EA) analgesia in formalin mice. EA was applied to ST 36 and GB 34 for 30 min before the formalin injection. The ST2 antibody was given either subcutaneously (A, B) or intrathecally (C, D) just before EA treatment, and paw lifting (A, C) and licking (B, D) times were observed. Data are presented as the mean ± S.E.M. *p < 0.05 vs. the Formalin+EA+IgG group.

To explore the effect of blocking IL-33/ST2 signaling on EA analgesia, the ST2 antibody was administered subcutaneously (5 μg) or intrathecally (10 ng) 30 min before EA treatment. Subcutaneous injection of the ST2 antibody significantly decreased the paw lifting and licking time in the EA treatment mice compared with the Formalin+EA+IgG group (P < 0.05) (Fig 4A and 4B). Additionally, intrathecal administration of the ST2 antibody markedly reduced paw lifting time in the second phase (P < 0.05) (Fig 4C and 4D). The results suggested that ST2 antibody administration could potentiate the EA analgesic effect in the formalin mice.

### Attenuation of the up-regulation of spinal IL-33 and ST2 after formalin injection

**Fig 5:**
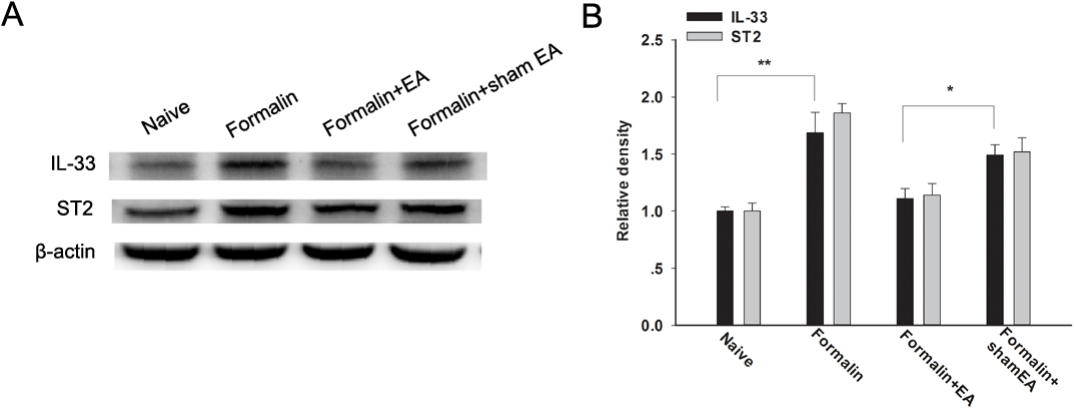
Expression of spinal IL-33 and ST2 after electroacupuncture (EA) treatment in formalin rats. EA was applied to ST 36 and GB 34 for 30 min before formalin injection. Representative bands are shown in A; the density of IL-33 and ST2 (B) were normalized against β-actin levels and expressed as a fold increase. Data are presented as the mean ± S.E.M. (n = 4 for each group). * p < 0.05, ** p < 0.01.

The expression of IL-33 and ST2 following formalin injection and EA treatment was investigated using western blot analyses. Compared with Naïve group, the expression of spinal IL-33 and ST2 were significantly increased after formalin injection (P < 0.05) (Fig 5A and 5B). The EA treatment for 30 min remarkably reduced the up-regulation of IL-33 and ST2, compared with formalin group (P < 0.05) (Fig 5A and 5B). However, no significant change was found in the sham EA group.

### Suppression of spinal MAPK phosphorylation by EA treatment in formalin mice

**Fig 6:**
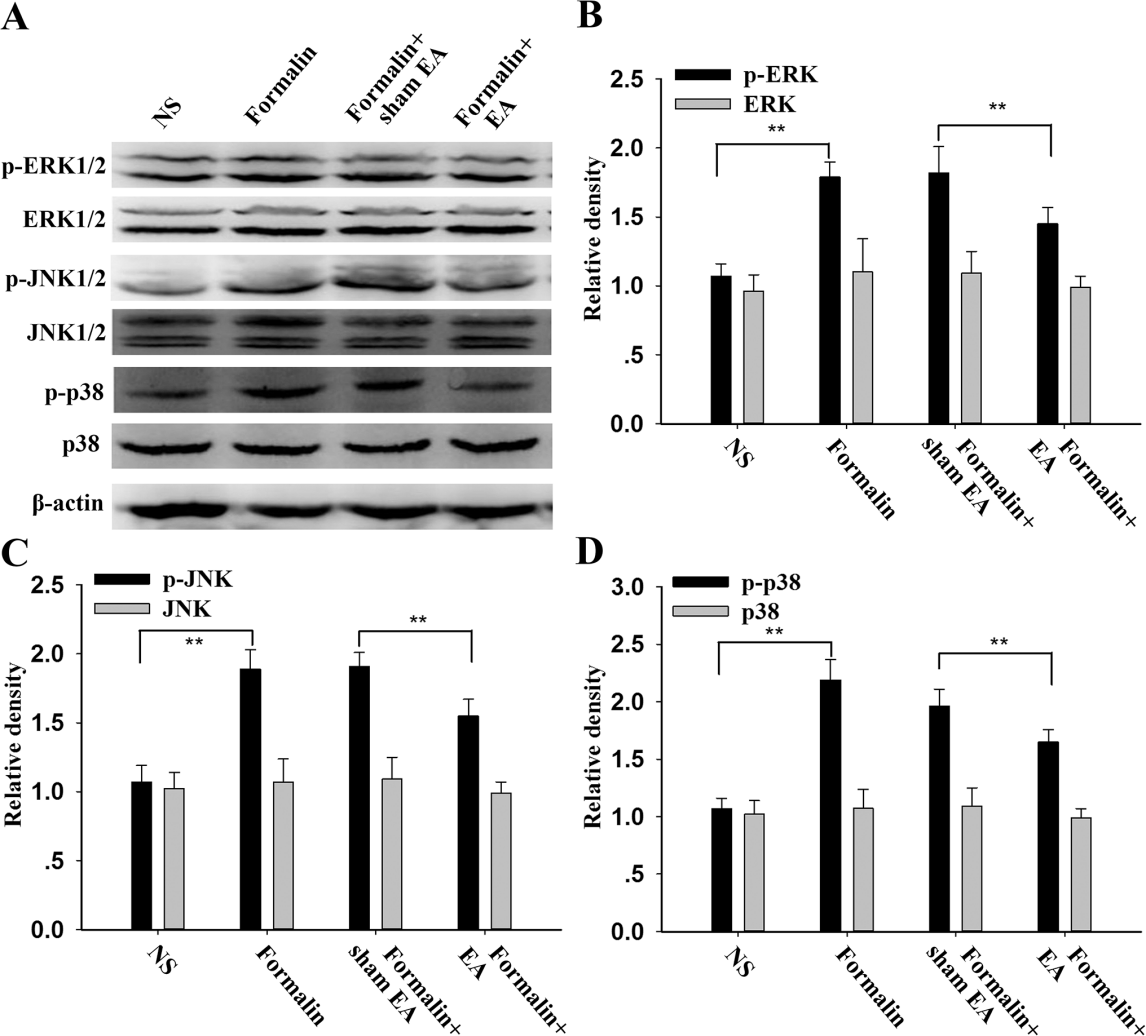
Expression of spinal MAPKs after electroacupuncture (EA) treatment in formalin rats. EA was applied to ST 36 and GB 34 for 30 min before the formalin injection. A shows the representative bands; the density of p-ERK and ERK (B); p-JNK and JNK (C); and p-p38 MAPK and p38 MAPK (D) levels were normalized to β-actin levels and expressed as a fold increase. Data are presented as the mean ± S.E.M. (n = 4 for each group). * p < 0.05, ** p < 0.01.

To examine the downstream pathway of IL-33/ST2 signaling in EA analgesia, we first observed the activation of MAPK signaling following EA treatment. After formalin injection for 45 min, expression of p-ERK, p-JNK and p-p38 MAPK were all remarkably increased in the lumber spinal cord compared with the NS group, whereas the total protein showed no significant change (Fig 6). EA treatment for 30 min markedly inhibited the increased expression of phosphorylated ERK, JNK and p38 MAPK induced by formalin injection compared with the sham EA group (P < 0.01).

### Effect of IL-33/ST2 pathway modulation on the EA treatment-mediated phosphorylation of spinal ERK and JNK in formalin mice

**Fig 7:**
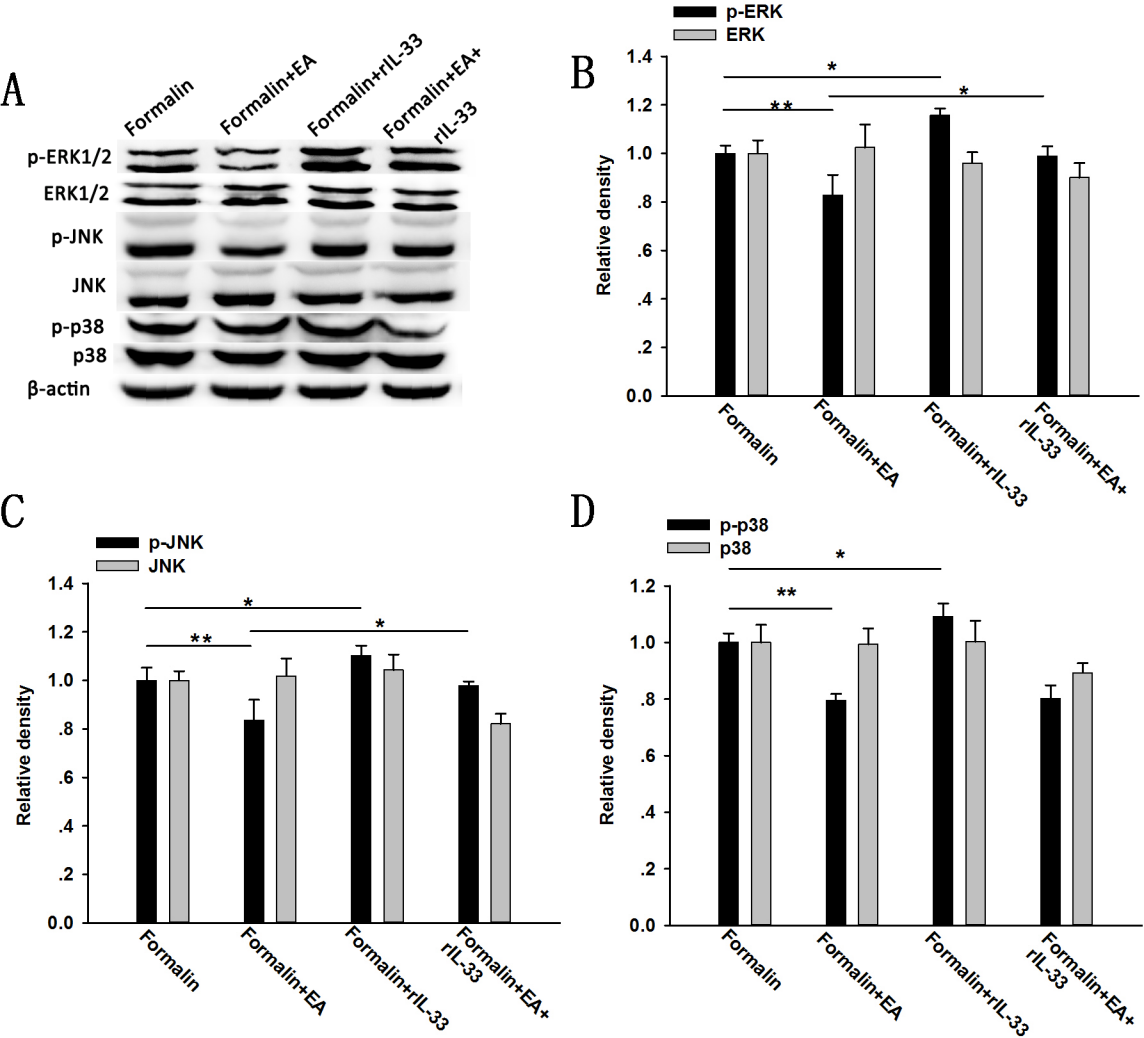
Expression of spinal MAPKs after electroacupuncture (EA) treatment and/or subcutaneous recombinant IL-33 (rIL-33) administration in formalin rats. EA was applied to ST 36 and GB 34 for 30 min before the formalin injection. Recombinant IL-33 was given subcutaneously immediately before EA treatment. Representative bands (A) and quantification of p-ERK and ERK (B); p-JNK and JNK(C); and p-p38 MAPK and p38 MAPK (D) is shown. Data are presented as the mean ± S.E.M. (n = 4 for each group). * p < 0.05, ** p < 0.01.

Next, we further examined the role of MAPK signaling in the IL-33/ST2 signaling in EA analgesia. Compared with formalin group, EA significantly inhibited the increased expression of phosphorylated ERK, JNK and p38 MAPK induced by formalin injection (P < 0.05), while subcutaneous rIL-33 administration further enhanced the increase (P < 0.05) (Fig 7). Recombined IL-33 injection remarkably attenuated the down-regulation of p-ERK and p-JNK induced by EA treatment compared with the Formalin+EA group (P < 0.05) (Fig 7), while no significant changes were observed in the expression of p-p38 MAPK. Total MAPKs, including ERK, p38 MAPK and JNK, were not changed by any treatment (P > 0.05).

**Fig 8:**
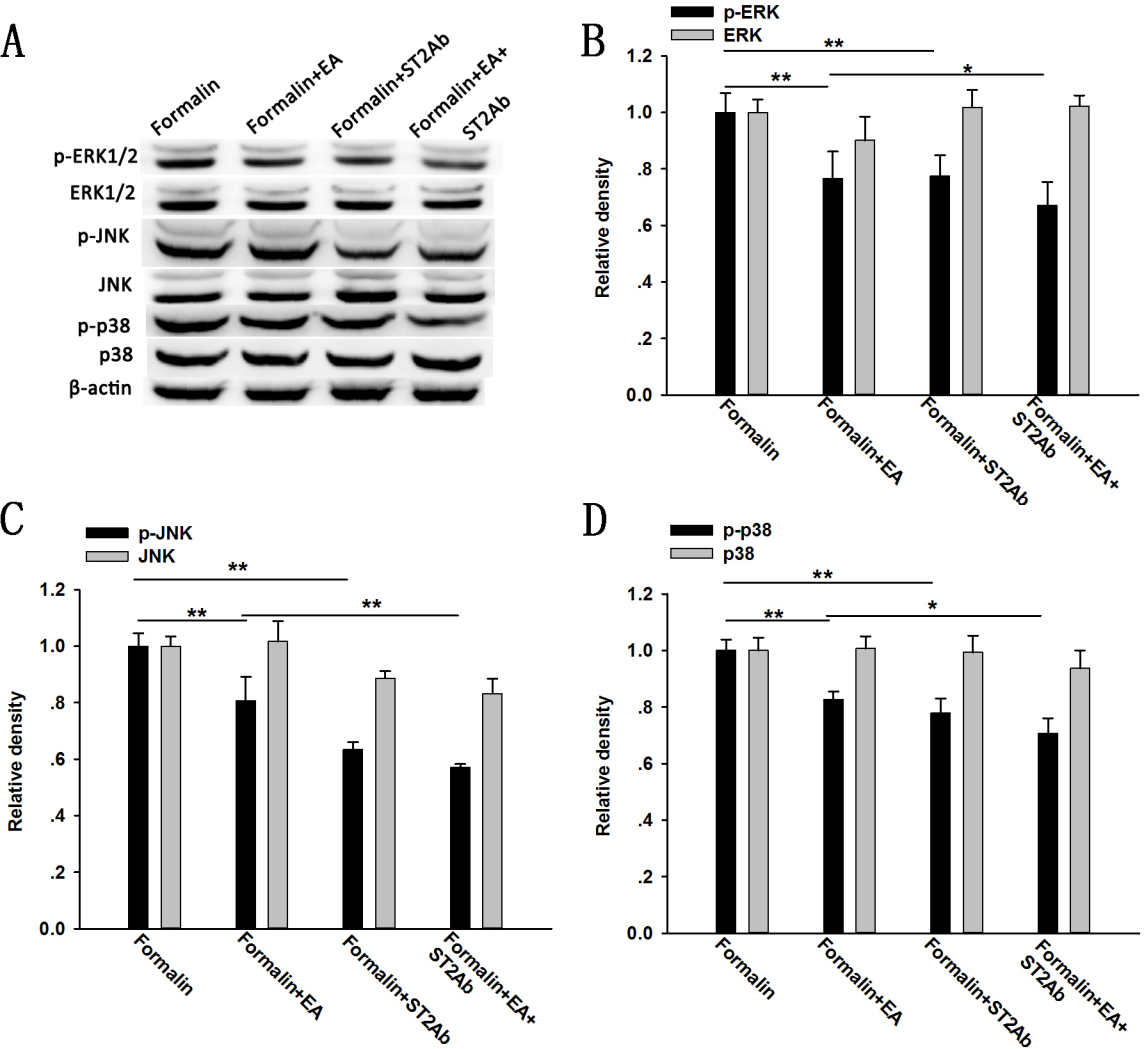
Expression of spinal MAPKs after electroacupuncture (EA) treatment and/or subcutaneous anti-ST2 antibody (ST2 Ab) administration in formalin rats. EA was applied to ST 36 and GB 34 for 30 min before the formalin injection. The ST2 Ab was administered subcutaneously immediately before EA treatment. Representative bands (A) and quantification of p-ERK and ERK (B); p-JNK and JNK (C); and p-p38 MAPK and p38 MAPK (D) is shown. Data are presented as the mean ± S.E.M. (n = 4 for each group). * p < 0.05, ** p < 0.01.

In addition, subcutaneous injection of the anti-ST2 antibody significantly inhibited the increased expression of phosphorylated ERK, JNK and p38 MAPK induced by formalin injection when compared with the formalin group (P < 0.05) (Fig 8). Anti-ST2 antibody administration markedly decreased the down-regulation of p-ERK, p-p38 MAPK and p-JNK induced by EA treatment compared with the Formalin+EA group (P < 0.05) (Fig 8). However, total ERK, p38 MAPK and JNK, were not changed (P > 0.05).

## Discussion

The present study demonstrated that the analgesic effect of EA on formalin-induced inflammatory pain can be attenuated by rIL-33 pretreatment and potentiated by neutralizing ST2 antibody administration, either subcutaneously or intrathecally. Additionally, the effect of IL-33 blockade on EA analgesia is through the activation of the downstream ERK and JNK signaling pathways.

In the present study, we used a classical inflammatory pain model based on the intra-plantar injection of formalin, although the procedures of the test has not yet been standardized, and variations such as the animal species used, the mice or rats strain used, housing conditions, and early social experience can influence the behavioral response. The formalin test had been widely used as an inflammatory pain model due to the following advantages over other pain models: (1) it induces behaviors that can be easily observed in a freely moving unrestrained animal [30]; (2) spontaneous pain-related responses can be observed in the absence of overt stimulation; that is, no additional stimulus is required to evoke nocifensive behaviors once injected, which better mimic clinical pain assessment in humans; (3) two phases can be observed in formalin pain, and the mechanisms behind the two phases are considered to be different: the early phase (0–5 min) of pain the response after formalin injection is due to the direct injury of tissues, reflecting nociceptive pain, whereas the late phase (10–45 min) is due to peripheral inflammation and central sensitization [30,31]. Furthermore, two different spontaneous nociceptive responses, i.e., paw licking and paw lifting, were monitored and used as parameters of pain intensity, similar to many previous studies [32]. As indicated in previous studies, paw lifting behavior represent the outcome of peripheral or spinal mechanisms related to the sensory component of pain, whereas paw licking behavior also has a supra-spinal component in addition to reflecting changes in the periphery and spinal cord [32].

Acupuncture and EA have been accepted as complementary and alternative medicines worldwide especially for the treatment of acute and chronic pain [33,34]. Here, we showed that EA treatment for 30 min decreased paw lifting and licking times in the two phases of pain in the formalin mice, which is in accordance with previous reports [32,35], suggesting acupuncture mediated the inhibition of both the peripheral and central sensitization. However, we can see from Fig 2 that paw licking time was remarkably decreased after EA administration in the early phase, but there was only a tendency toward a decrease in the late phase. This result indicates the supra-spinal component maybe less important in EA analgesia on formalin pain. Several processes have been proposed to explain the mechanisms of EA analgesia, and results have indicated that acupuncture dampens the transmission of noxious inputs at the spinal level with the involvement of spinal opioids, serotonin, norepinephrine, glutamate, glia/cytokines, and signaling molecules [4,5,36]. Previous research also emphasized the important role of local adenosine A1 receptors in the anti-nociception of acupuncture [7]. Previous studies, including some studies from our laboratory, showed that interleukins, such as IL-1β, are modulated in EA analgesia in inflammatory pain [8,37]. Consistently, we showed here that IL-33, a novel IL-1 family member with a structure similar to that of IL-1β, also played an important role in EA analgesia in acute inflammatory pain. To the best of our knowledge, this is the first study examining a relationship between IL-33 and acupuncture analgesia.

Specifically, we found that the analgesic effect of EA on formalin pain behaviors was attenuated by the subcutaneous or intrathecal injection of rIL-33 and was potentiated by the administration of the ST2 antibody. In addition, the expression of IL-33 and ST2 protein in the spinal cord was significantly increased after formalin injection, and EA treatment reduced the up-regulation. Combined with our previous results that the potentiating effect of IL-33 on formalin pain was significantly blocked or even eliminated by the ST2 antibody [18], we can conclude that the analgesic effect of EA may be attributed to the inhibition of IL-33 and ST2 expression and may be further affected by IL-33/ST2 signaling. Furthermore, in addition to the spinal cord, the IL-33 in the local plantar region or in the dorsal root ganglion (DRG) may also play a role in this process. This possibility needs further investigation because we observed distinct mRNA and protein expression of IL-33 and ST2 in the DRG (data not shown).

IL-33 interacts with the receptor complex containing ST2 and IL-1RAcp and leads to the recruitment of the myeloid differentiation primary-response protein 88 (MyD88), IL-1R-associated kinase 1 (IRAK1) and IRAK4 to the receptor complex, which, in turn, results in the activation of NF-κB, MAPKs and JAK2 [12,21]. A previous report indicated that formalin-induced inflammatory pain is accompanied by the activation of peripheral and spinal MAPKs [38,39]. Consistent with this idea, the present study showed that after formalin injection, the expression of phosphorylated components of the MAPK pathway, including p-ERK, p-p38 MAPK and p-JNK, was remarkably increased in the spinal cord. Additionally, we found that EA treatment could inhibit the phosphorylation of ERK, p38 MAPK and JNK, which is also consistent with previous studies [23,24]. Also, from Figs 7 and 8, we can see that subcutaneous rIL-33 administration further enhanced the formalin induced up-regulation of ERK, p38 MAPK and JNK phosphorylation, whereas the anti-ST2 antibody inhibited this up-regulation, indicating the involvement of MAPK signaling in the IL-33/ST2 mediated formalin pain. Administration of the anti-ST2 antibody could further decrease the down-regulation of p-ERK, p-JNK, and p-p38 MAPK, induced by EA. However, IL-33 pretreatment could only attenuate the EA induced down-regulation of p-ERK and p-JNK in the formalin mice, but not p-p38 MAPK induced by EA. hese results suggest that the blocking effect of IL-33 on EA analgesia is probably due to the activation of ERK and JNK signaling.

Our recent study demonstrated that spinal IL-33 is predominantly located in astrocytes [19], whereas the distribution of ST2 in the central nervous system is disputed. ST2 has been reported to be expressed in astrocytes, but not in microglia or neurons in the brain [40]. ST2 has also been reported to be expressed in both astrocytes and microglia [41] and in neurons [42] in the murine spinal cord. Using immunochemistry as well as the Fluorescence In Situ Hybridization (FISH) combined immunohistochemical technique, we clearly showed that ST2 is mainly distributed in neurons and astrocytes, and not in microglia in the mouse spinal cord (data not shown). In addition, IL-1RAcp is reported to be expressed in neurons, astrocytes and microglia [41]. Therefore, we can speculate that IL-33 is released from the astrocyte and then acts on astroglial or neuronal ST2 receptor in an autocrine or paracrine manner during pain. In addition, it has been reported that ERK and JNK are mainly distributed and activated in the astrocytes and neurons, whereas p38 MAPK is mainly distributed and activated in microglia [43]. Thus, it is reasonable to assume that IL-33 blocked EA analgesia by activating ERK and JNK signaling in spinal astrocytes or neurons, not by activating microglial p-38 MAPK. These results were also in accordance with a previous report that the analgesic effect of acupuncture is mediated via the inhibition of JNK activation in astrocytes after spinal cord injury [23].

In addition, the potentiating effect of the ST2 antibody on the analgesic effect of EA suggests a possible new way of enhancing EA analgesia by combining the treatment with IL-33/ST2 pathway blockage, which could address the drawback of the inadequate analgesic effect of EA in clinical practice. We also found that the analgesic effect of the ST2 antibody on formalin pain was not through its actions on the opioid receptor because the analgesic effect of the ST2 antibody was not affected by naloxone (data not shown). Thus, the additive analgesic effect of the ST2 antibody on EA is not due to potentiated activation of the opioid system. Using western blotting, we found an additional inhibitory effect of the ST2 antibody on the suppression of the down-regulation of p-ERK induced by EA, suggesting the potentiating analgesic effect of the ST2 antibody on EA is possible through further inhibition of the ERK pathway.

Taken together, these data indicate that the analgesic effect of EA on formalin-induced inflammatory pain in mice is mediated by inhibition of IL-33/ST2 signaling and the subsequent downstream ERK and JNK pathways. The present study may initiate a discussion on the possible roles of IL-33/ST2 signaling in the therapeutic effects of acupuncture on various diseases.
